# Optimizing human pulmonary perfusion measurement using an in silico model of arterial spin labeling magnetic resonance imaging

**DOI:** 10.14814/phy2.14077

**Published:** 2019-06-13

**Authors:** Daniel A. Addo, Wendy Kang, Gordon Kim Prisk, Merryn H. Tawhai, Kelly Suzzane Burrowes

**Affiliations:** ^1^ Auckland Bioengineering Institute University of Auckland Auckland New Zealand; ^2^ Departments of Medicine and Radiology University of California San Diego La Jolla California; ^3^ Department of Chemical and Materials Engineering University of Auckland Auckland New Zealand

**Keywords:** Arterial spin labeling, magnetic resonance imaging, pulmonary blood flow, regional pulmonary blood

## Abstract

Arterial spin labeling (ASL) magnetic resonance imaging (MRI) is an imaging methodology that uses blood as an endogenous contrast agent to quantify flow. One limitation of this method of capillary blood quantification when applied in the lung is the contribution of signals from non‐capillary blood. Intensity thresholding is one approach that has been proposed for minimizing the non‐capillary blood signal. This method has been tested in previous in silico modeling studies; however, it has only been tested under a restricted set of physiological conditions (supine posture and a cardiac output of 5 L/min). This study presents an in silico approach that extends previous intensity thresholding analysis to estimate the optimal “per‐slice” intensity threshold value using the individual components of the simulated ASL signal (signal arising independently from capillary blood as well as pulmonary arterial and pulmonary venous blood). The aim of this study was to assess whether the threshold value should vary with slice location, posture, or cardiac output. We applied an in silico modeling approach to predict the blood flow distribution and the corresponding ASL quantification of pulmonary perfusion in multiple sagittal imaging slices. There was a significant increase in ASL signal and heterogeneity (COV = 0.90 to COV = 1.65) of ASL signals when slice location changed from lateral to medial. Heterogeneity of the ASL signal within a slice was significantly lower (*P = *0.03*)* in prone (COV = 1.08) compared to in the supine posture (COV = 1.17). Increasing stroke volume resulted in an increase in ASL signal and conversely an increase in heart rate resulted in a decrease in ASL signal. However, when cardiac output was increased via an increase in both stroke volume and heart rate, ASL signal remained relatively constant. Despite these differences, we conclude that a threshold value of 35% provides optimal removal of large vessel signal independent of slice location, posture, and cardiac output.

## Introduction

Arterial spin labeling magnetic resonance imaging (ASL MRI) (Hopkins et al. 2005; Bolar et al. 2006; Henderson et al. 2009) is a technique used to measure blood flow by using the water in blood as an endogenous tracer. This method does not expose the subject to ionizing radiation and does not require any contrast agents providing an extremely safe and repeatable measure of regional perfusion. The ASL MRI technique works by obtaining two cardiac‐gated MR images during a single breath‐hold. The two images are acquired in the same way but are prepared using different MR tagging protocols. In the first image, a slice‐selective radiofrequency (RF) inversion pulse is applied during diastole such that the signal within an imaging slice is reduced. The image is acquired after a time interval, TI, corresponding to 80% of a healthy subject's R‐R interval and appears bright due to the signal from the volume of blood with full MR signal that has entered the slice during systole; this is termed the blood “bright” image. The second image involves the application of a nonselective, or global, inversion RF pulse such that the signal in the entire lung is reduced. After the same time interval, the image is acquired and because all blood has its signal reduced this produces a “dark” image. A subtraction between the “bright” and “dark” images removes the stationary lung tissue and produces the ASL image which quantifies delivery of blood to the imaging slice from a single systolic ejection.

Originally developed to measure cerebral blood flow (Aslan and Lu 2010; Detre et al. 2012; Koretsky 2012) adaptation of this methodology to measure regional pulmonary perfusion includes unwanted signal. While this technique aims to quantify local perfusion, in reality the magnetic resonance (MR) image will also contain signal from blood within larger pulmonary arterial and pulmonary venous (non‐capillary) vessels (also called conduit vessels). Thus, ASL images of the lung consist of both blood delivered to, or destined for, the capillary bed in the imaged slice (the wanted signal) and blood that was delivered to the conduit vessels within the slice but destined for other parts of the lung (the unwanted signal). Previously, there have been two main approaches developed to remove this unwanted signal: (1) intensity thresholding, a method that removes voxels with the highest signal intensities under the assumption that these voxels are comprised of conduit vessel signal (Burnham et al. 2009; Henderson et al. 2009; Burrowes et al. 2012) and (2) a statistical clustering (bivariate) approach that utilizes both the bright and dark MR images to remove conduit vessel signal contribution (Walker et al. 2015).

The main image post‐processing approach that has been used to minimize the amount of non‐capillary signal in an ASL MR image is “intensity thresholding” (Henderson et al. 2009; Burrowes et al. 2012). This method consists of removing voxels above a certain signal intensity using the knowledge that the brightest or highest intensity voxels will signify large conduit vessels. This provides a fast and computationally efficient method to remove or filter conduit signal from the image. A previous study by Burrowes et al. 2012 suggested that an optimal filtering threshold of 35% would remove the majority of large vessel signal without sacrificing the capillary signal. However, this study considered only a single sagittal slice in the supine posture at a single cardiac output. It is not clear whether this single threshold is optimal across varying conditions including different postures (prone versus supine), different cardiac outputs, and whether this threshold should change depending on which location is being imaged.

The current study addresses this by using an in silico model of the human pulmonary circulation (Clark et al. 2011; Burrowes et al. 2012) to simulate the ASL MRI protocol and resultant image (Burrowes et al. 2012) of a healthy human lung. The relevance of the threshold analysis outlined in this study is to optimize the accuracy of the perfusion measurement in vivo using ASL MRI method for human studies. To do this, we assessed the sensitivity of both the gradient and COV of the ASL signal to thresholding and compared them to the gradient and COV of the underlying perfusion of the reference model. This enabled the prediction of threshold values that served to best match the ASL signals to the underlying perfusion signals. We have also incorporated variations in heart rate, posture and slice location to assess how sensitive the measurement outcomes are to such variations that may be present in various imaging scenarios.”

## Methods

### Study overview

This study involved the use of an anatomically based in silico model of the pulmonary circulation (Burrowes et al. 2005) within which equations representing flow to determine regional pressures and velocities within each blood vessel and capillary bed in the model (Clark et al. 2011) were solved. To simulate the ASL image, the creation of MR signals as would occur in the ASL MRI technique was replicated. Specifically, an image slice through the lung model was defined and all blood within the specified imaging slice determined. Using the velocity values for each “portion” of blood (see Blood flow model section), the location of that blood at the time of tagging (by tracking backwards through the network) was determined in order to identify the amount of magnetization (signal) that each “portion” of blood will have at the time of imaging within the image slice. This gives an in silico representation of the amount of signal in the imaging plane during imaging (Burrowes et al. 2012).

### Blood flow model

The human lung model geometry used in this study was obtained from a healthy male (age: 23 years, weight: 80.9 kg, height: 1.87 m) and has formed the basis of previous studies (Burrowes et al. 2005; Clark et al. 2010, 2011). To simulate blood flow within the full pulmonary network, cardiac outputs of 4, 5, 6, and 8 L/min were applied at the pulmonary trunk as the first boundary condition and a left atrial pressure of 5 mmHg applied as the outlet boundary condition. Flow of blood in each element of the model geometry was determined based on the blood vessel type (i.e., as artery, vein, arteriole, venule, or capillary). Blood flow within the arteries and veins were modeled using the 1D Poiseuille equation and the effects of gravity (assumed to be acting on the blood in the direction of the vessel centerline). Because the lengths of the arterioles and venules were assumed to be sufficiently small, the effects of gravity on the blood were neglected and blood flow through these vessels modeled using only the 1D Poiseuille equation. By using the 1D model, flow was assumed to dominate along the centerline of the vessel. Furthermore, the sheet flow model of Fung and Sobin (1969) was used to describe blood flow in the capillary sheet. The blood flow model used in this study has been validated and considered sufficiently accurate. The model also incorporates distension of arterial and venous vessels (via a pressure–area relationship) and recruitment of capillaries, via the sheet flow model of Fung and Sobin. Further details on how blood flow was modeled within the pulmonary circuits can be found in (Clark et al. 2011). Blood flow was simulated at FRC in the supine position, and then prone position by reversing the direction of gravity. While blood flow was simulated in the whole lung, in the subsequent ASL simulation, only the right lung was used due to the predominant use of the right lung for imaging studies (Prisk et al. 2008; Arai et al. 2009; Burnham et al. 2009; Henderson et al. 2009).

### In silico ASL MRI simulation

**Figure 1:**
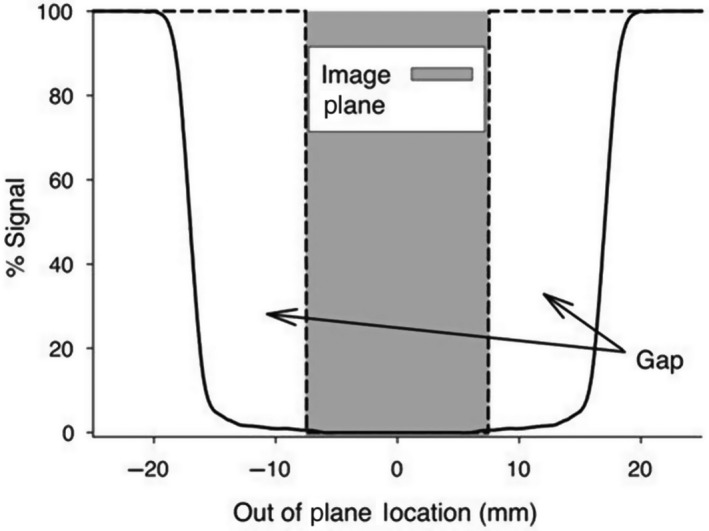
Characterization of the labeling profile based on a typical inversion band taken from a Bloch equation simulation of the inversion plane used in an ASL experiment (Hopkins et al. 2007a). Note, the inversion plane is greater than the imaging plane (~15 mm) creating what is known as the inversion gap. An ideal inversion plane is illustrated with the black dashed line exactly matching the boundaries of the image plane. Figure used with permission from (Burrowes et al. 2012).

To infer the ASL MR image from the blood flow solution, the ASL MRI protocol was replicated using a previously developed ASL model (Burrowes et al. 2012). In modeling the ASL signal associated with each blood element, three parameters that were relevant were the velocity profile and blood volume of each in‐plane blood vessel as well as the inversion pulse. The velocity profile and blood volumes were obtained from a previously validated and sufficiently accurate blood flow model which has been used in previous studies (Clark et al. 2010, 2011). While the inversion pulse profile has been used in an ASL experiment (Hopkins et al. 2007a) and thus considered reliable and accurate in this study. Furthermore, this study did not involve any real imaging data. By using only the blood flow solution within the vessels and avoiding any static tissue, the ASL setup was simplified and the influence of coil inhomogeneity with depth did not need to be considered. Because only pulmonary blood flow was represented in the model (i.e., no other lung water/tissue was included as would be present in reality), only the blood “bright” image protocol was simulated and assumed to be representative of the ASL MRI difference image (blood “bright” minus blood “dark” image). As mentioned earlier, the “bright” image is created by applying a spatially selective inversion pulse that inverts the magnetization of the tissue within a volume encompassing, but slightly thicker than, the image plane (Fig. 1). Ideally, the inversion would only be applied to the image slice (dashed line in Fig. 1); however, the MRI scanner in unable to produce this ideal inversion pulse. The volume of inversion outside of the image plane is termed the inversion gap and results in incomplete tagging of blood within this region. As a result, two different equations (eqs. (1) and (2)) were used, depending on the location of the blood during tagging, to determine the ASL signal. Equation (1) is used if blood elements originate from within the inversion gap because the signal they contribute to the imaging plane is highly dependent on their location within the inversion gap. Equation (2) was used to model blood originating from outside of the inversion gap. Only a healthy subject is considered in this study hence TI corresponding to 80% of RR was adequate. Furthermore, there is no interplay between the individual components of the model in terms of the simulated ASL signal.

(1) $$S_{\mathrm{BW}}=\left[\left[M_{\mathrm{OB}}-\left(M_{\mathrm{OB}}-\text{Mo}\left(0\right)\right)e^{\frac{-TI}{T1}}\right]*e^{\frac{-\mathrm{TE}}{T2}}\right]*V_{B}$$

(2) $$S_{\mathrm{BO}}=M_{\mathrm{OB}}*e^{\frac{-\mathrm{TE}}{T2}}*V_{B},$$

where *S*_BW_ is the signal of blood within the inversion gap, *S*_BO_ is the signal of blood outside the inversion gap, Mo(0) is the fraction of longitudinal magnetization in relation to the equilibrium longitudinal magnetization at time *t* = 0 defined by the distribution in Figure 1 (Bloch curve), M_OB_ is the equilibrium longitudinal magnetization with a value of 1, *T*1 is the longitudinal relaxation time, *T*2 is the transverse relaxation time, and TE is the echo time. These equations are based on those presented by Henderson et al. (2009). In this study values of magnetic field strength (Bo) = 1.5 T, *T*1 = 1300 msec, *T*2 = 254 msec, TE = 36 msec (values from(Bolar et al. 2006)) were used and voxel dimensions used were 15 mm × 3 mm × 1.5 mm (67.5 mL); consistent with typical voxel dimensions used in ASL MRI studies (Bolar et al. 2006; Henderson et al. 2009). VB is the blood volume per vessel element within the slice of interest. ASL signal in mL/min/cm^3^ was estimated by dividing the volume of blood that has flown into a voxel (for a given TI) by the volume of blood in a fully filled voxel.

Calculation of the in silico ASL image consists of the following steps:

1. Define the image slice through the model, in this study we simulate five 15 mm sagittal slices which covers most of the right lung;
2. For a given TI, we use the model‐predicted velocity values of in‐plane blood elements to determine where the blood was at the time of tagging (tracing backward through the network model and using the fact that distance = velocity/time);
3. Equations (1) and (2) are used to determine the amount of signal that will have been applied to each portion of blood. If the blood was within the inversion gap at the time of tagging we apply equation (1), if the blood was outside of the inversion gap we apply equation (2).

Additional details of how the ASL computational model works can be found in (Burrowes et al. 2012).

### Investigating the impact of varying cardiac output on intensity thresholding

To assess whether the intensity thresholding value to remove conduit signal should be altered as a function of variation in cardiac output (CO), CO was varied by increasing the heart rate (HR) and/or stroke volume (SV) (where CO = HR × SV). Because the blood flow model used for this study is steady state (it does not include time‐dependence), SV change was represented by change in pulmonary artery flow rate, and HR change was represented by altering the inversion time, TI. Variation in TI alters the location of the blood during tagging and results in a different magnetization profile being applied to the blood. Four combinations each of SV and HR were used, as listed in Table 1.

**Table 1:** Parameters used to investigate variation in cardiac output (CO), noting that CO = heart rate (HR) × stroke volume (SV)

	Scenario 1 Varying SV			Scenario 2 Varying HR			Scenario 3 Constant CO			Scenario 4 Varying HR and SV		
	SV	HR	CO	SV	HR	CO	SV	HR	CO	SV	HR	CO
i	0.066	60	4	0.083	48	4	0.104	48	5	0.050	80	4
ii	0.083	60	5	0.083	60	5	0.083	60	5	0.058	86	5
iii	0.100	60	6	0.083	72	6	0.069	72	5	0.067	90	6
iv	0.133	60	8	0.083	96	8	0.052	96	5	0.083	96	8

In scenario 1 changes in CO were achieved by altering SV with HR kept constant; in scenario 2 changes in CO were achieved by changing HR with SV kept constant; in scenario 3 CO was kept constant at 5 L/min while varying SV and HR; in scenario 4 changes in CO were achieved by changing both SV and HR.

NB/ SV, stroke volume (L/beat); HR, heart rate (BPM); CO, cardiac output (L/min). HR values implemented by changing inversion time (TI) as follows: HR = 48, 60, 72, 96 BPM corresponded to TI = 1000, 800, 667, 500 msec, respectively and HR = 80, 86, 90, 96 BPM corresponded to TI = 600, 558, 533, 500 msec, respectively.

### Determining the optimal intensity threshold value for removing conduit vessels

The variables presented in Table 2 were compared as the range of thresholding values (*x)* was investigated, starting from no thresholding/removal of signal (*x* = 100%) to removal of nearly all signal (*x* = 5%). The value of 100% corresponds to an absolute ASL value of 67.5 mL (this is the total voxel volume, meaning that the voxel was completely filled with blood signal) and the same 100% value was applied across all simulation studies.

**Table 2:** Description of variables used in the cost function (eq. (3), Fig. 2). Perfusion as applied in the table refers to model estimation of capillary perfusion

Variable	Description of variable
*Q*_fraction_ (%)	Perfusion fraction: Calculated as the ratio of the ASL signal arising from perfusion to the total ASL signal (arising from all blood) within the slice.
*Q*_remain_ (%)	% perfusion remaining: Calculated as the ratio of ASL signal arising from perfusion after thresholding to the ASL signal arising from perfusion prior to thresholding.
*C*_removed_ (%)	% conduit signal removed: Calculated as the ratio of ASL signal arising from conduit vessels after thresholding to the value prior to thresholding.
Δgrad (%)	% difference in gravitationally dependent gradient between the total ASL signal (perfusion, arterial and venous) and perfusion.
ΔCOV (%)	% difference in the coefficient of variation (COV) between the two datasets [ASL (perfusion, arterial and venous) and perfusion].

The cost function (*C*), shown in equation (3), was used to evaluate the best threshold to apply to optimize the balance between the variables in Table 2. The cost function (which is a mathematical operation) analyses only the output of the ASL model and is tested only in a healthy subject.

(3) $$C\left(x\right)=\left[1-Q_{\mathrm{frac}}\left(x\right)\right]+\left[1-Q_{\mathrm{remain}}\left(x\right)\right]+\left[1-C_{\mathrm{removed}}\left(x\right)\right]+\Delta \mathrm{cov}\;\left(x\right)+\Delta \text{grad}\left(x\right)$$

Equation (3) was applied to determine the optimal intensity threshold value for the different conditions we are simulating: comparing values in all five sagittal slices, in the prone and supine posture and with varying cardiac outputs (Table 2). Ultimately, we were trying to maximize the amount of conduit signal removed (*C*_removed_) and the amount of perfusion signal remaining in the image (*Q*_remain_), while minimizing the loss of signal from perfusion (reflected as *Q*_fraction_) while filtering. We also want to ensure that the ASL signal accurately describes the underlying perfusion in terms of the gravitationally dependent flow gradient (grad) and heterogeneity (*COV*) and thus include the parameters *Δ*grad (difference in gradient between perfusion and the ASL signal) and *Δ*COV (difference in heterogeneity between perfusion and the ASL signal).

## Results

### Determining the optimal intensity threshold value for removing conduit vessels

#### Sensitivity analysis of the intensity threshold optimization decision variables

**Figure 2:**
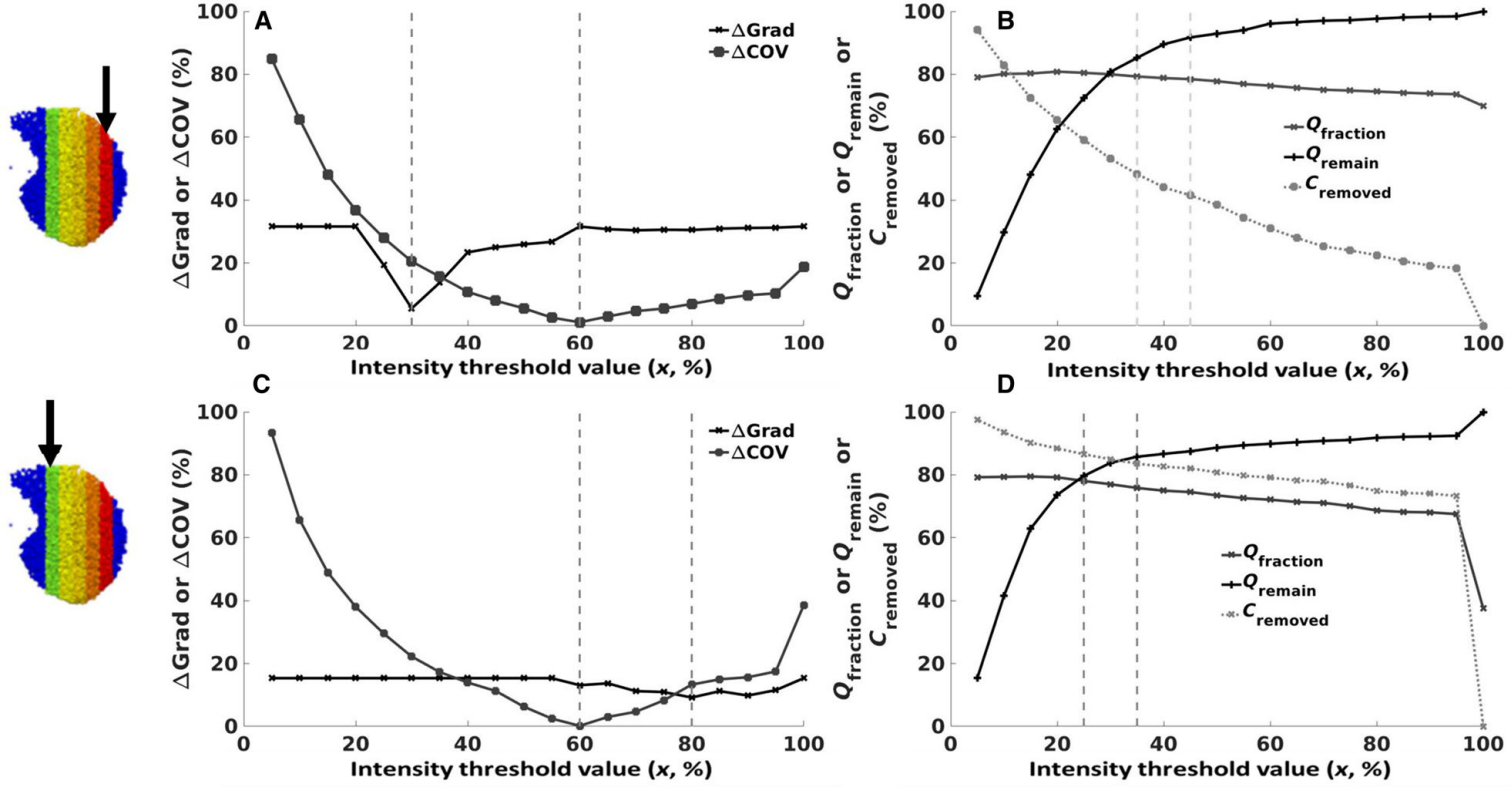
Plots demonstrating the impact of variation in the intensity thresholding value (*x*, %) as a function of the optimization cost function variables (*Q*_fraction,_ *Q*_remain,_ *C*_removed,_ Δgrad, ΔCOV, refer to Table 2 for definitions). Results are shown for a lateral slice (A, B – slice 1) and a medial slice (C, D – slice 5). The vertical dashed lines within each plot represent the range of ideal threshold values.

Figure 2 examines the effect of intensity thresholding (removing high‐intensity voxels dominated by non‐capillary blood signals) on the cost function variables. These plots demonstrate the impact of thresholding (starting with all ASL signal included, 100% and filtering out the highest intensity voxels in 5% increments) from 100% to 5%. Results are shown for a lateral (Fig. 2A and B) and medial slice (Fig. 2C and D). The five variables plotted are the ones included in the cost function (refer Table 2 for definitions). Note, that absolute values are displayed, so for example, when filtering goes below ~60% Figure 2A and C show an increase in ΔCOV, this is because the COV of the ASL values start to become smaller than the COV of the underlying perfusion.

Figure 2 shows that depending on which variable is being optimized and which slice is being looked at, there is a large variation in the ideal threshold value. For example, considering the lateral slice (Fig. 2A and B), the best representation of COV (ΔCOV*)* would be obtained when a threshold value of *x* = 60% was applied. Equally, to optimize the gradient difference alone (Δgrad), a threshold of *x* = 30% would be best. Considering other variables in this same way, *Q*_fraction_ *, Q*_remain_ *,* and *C*_removed_ (Fig. 2B), the ideal threshold values would be somewhere between 35% and 45%. To determine the best threshold for all variables combined, we added the values for each variable (see eq. (3) for the cost function) and considered the minimum value overall to be the best threshold value. For this lateral slice, the cost function predicted an optimal threshold value of 35%.

#### Do we need to change threshold as a function of slice?

**Figure 3:**
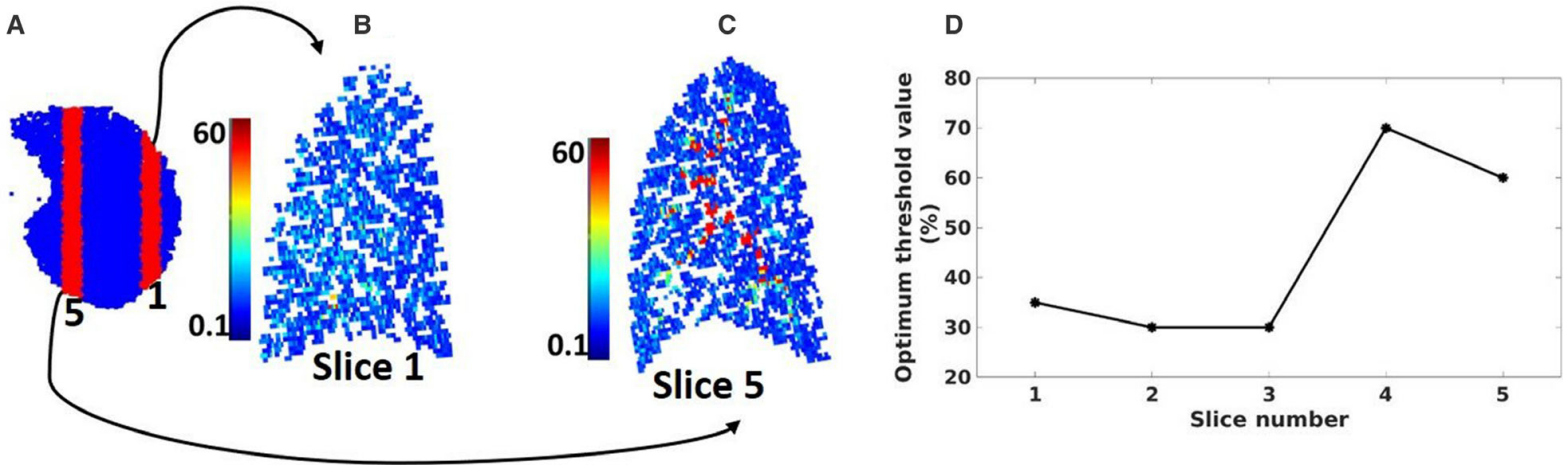
Figures showing ASL value (ml/min/mm^3^) in medial and peripheral slices (Fig. 3B and C, respectively). The more medial slice contains larger vessels and therefore greater ASL signal. Figure 3D shows the optimal threshold predicted using the cost function over each of the five sagittal slices.

Figure 3D below plots the optimal filtering threshold as predicted by the cost function for five sagittal slices covering most of the right lung. The location of slices 1 and 5 is displayed in Figure 3A and the remaining slices (2–4) lie within this range. The cost function determines a relatively large difference in the optimal filtering value to be used in the three lateral slices (~30%) compared to the value in the two medial slices (~60%, Fig. 3D). Figure 3B and C display the simulated ASL MR image for slices 5 and 1, respectively. Slice 5 (Fig. 3B) is a medial slice and contains several large conduit vessels; evidenced by the voxels in red with the highest ASL signal of >60 mL/min/mm^3^ (meaning the whole voxel, 67.5 mL, is full of blood). Slice 1 (Fig. 3C) is a lateral slice and does not contain any voxels with very high intensities.

**Figure 4:**
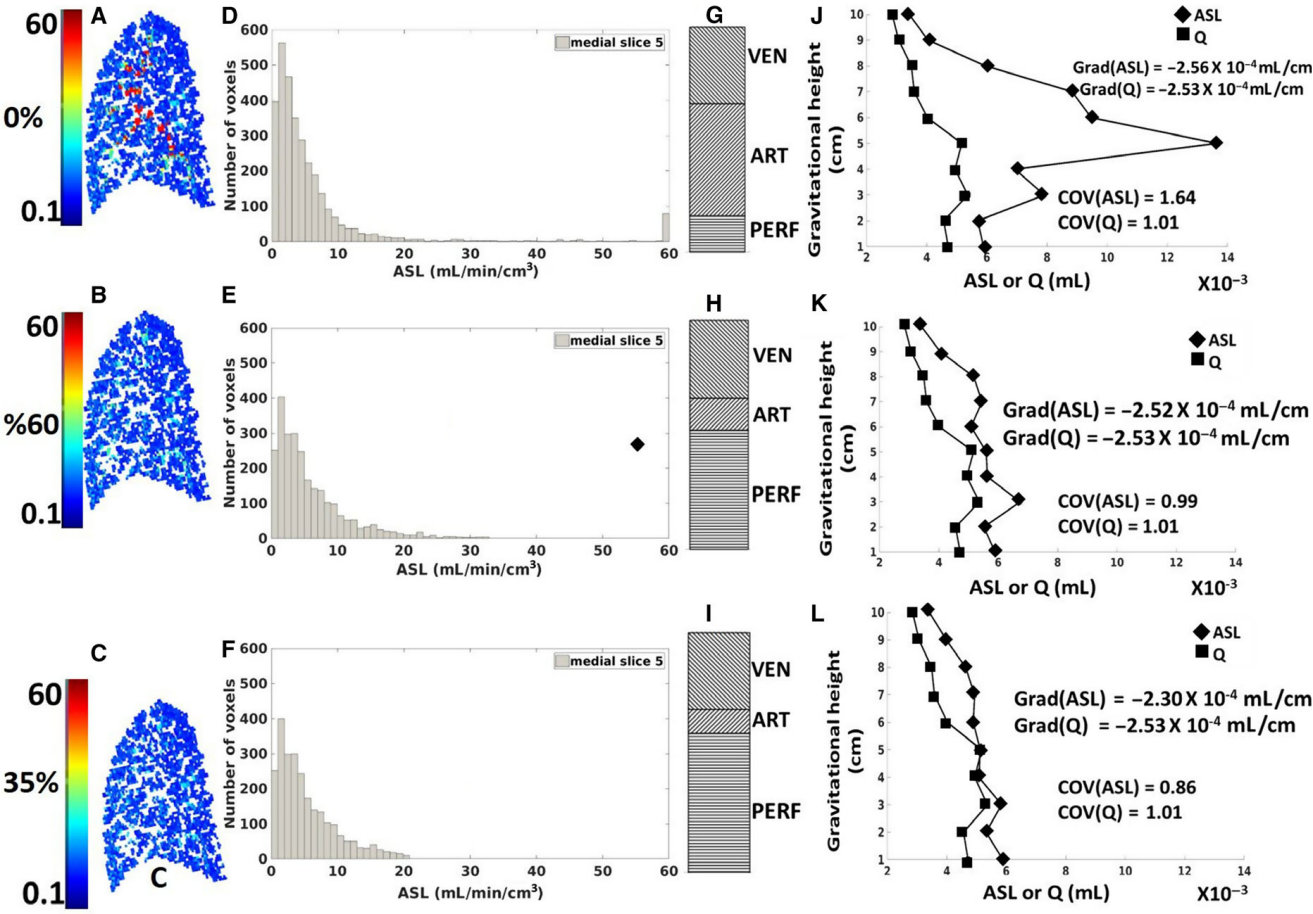
Demonstration of the impact of various thresholding (no thresholding, 60% and 35% thresholding) on the ASL representation of blood flow in a medial slice (slice 5). (A–C) In silico quantitative representation of the ASL image; (D–F) Frequency histogram of ASL signal; (G–I) Indication of proportion of signal from arteries, veins, and capillaries; (J–L) Plots of ASL signal and perfusion as a function of gravitationally dependent height. Values for the linear gradient fitted to the gravitational distribution of blood flow for ASL and perfusion (Grad(ASL), Grad(Q)) and values for the coefficient of variation for ASL and perfusion (COV(ASL), COV(Q)) are included.

**Figure 5:**
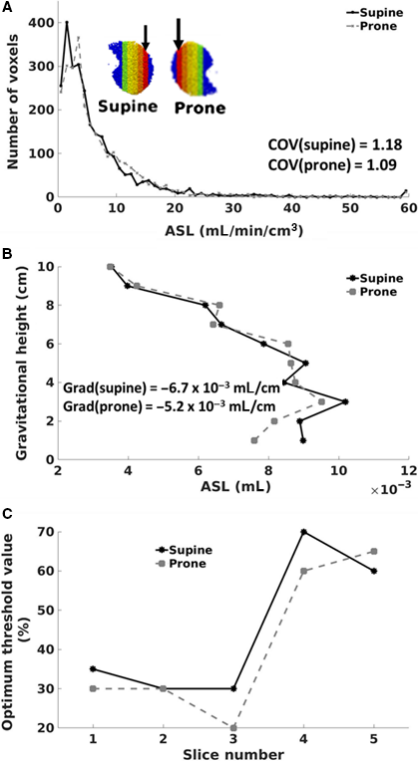
(A) Frequency histogram of ASL signal in slice 1 for both prone and supine posture; (B) Plot of ASL signal a function of gravitationally dependent height for both prone and supine posture; C: Plots of optimal threshold values predicted using the cost function over each of the five sagittal slices for both prone and supine postures.

Figure 4 presents the impact of thresholding on the various outcome parameters from the ASL measurement. The impacts of no thresholding (100%) and of thresholding to 60% (as proposed by the cost function) and 35% (the current thresholding standard) were demonstrated on a medial slice (slice 5). Figure 5A–C shows the ASL distribution within the slice. The hot spots in Figure 4A show regions in the slice with flows dominated by large vessels; these were removed after thresholding using a value of 60% (Fig. 4B). Figure 4D–F demonstrates the frequency histograms of the simulated ASL measurement. The histograms were plotted using a bin size of 1 mL/min/cm^3^. The simulated ASL histograms compare well with distributions from MRI measurements (see for example Figure 4 in Hopkins et al. (2007b)), showing a peak value ~2 mL/min/cm^3^ and a long tail indicating regions with higher flows dominated by conduit vessels.

This is clearly seen in slice 5 which includes several large conduit vessels. It can be seen that as the highest intensity voxels are removed by thresholding the proportion of perfusion signal increased from around 13% to 52% and then 63% when filtering to 60% and 35%, respectively. Figure 4J–L shows the gravitationally dependent distribution of flow through the sagittal image plane in the supine flow model, moving along the dorsoventral axis (height = 1, dorsal; height = 10, ventral). In Figure 4J, the ASL quantification of flow exhibits larger values than perfusion, particularly in the mid region of the lung, due to the contribution of conduit signal. After filtering (Fig. 4K and L) the ASL values more closely represent the perfusion distribution, both in terms of the absolute value and the linear fitted gradient of gravitationally dependent flow, although the gradient values are fairly well‐matched in all representations.

COV values and linear gradient values are included in Figure 4J–L. Prior to thresholding, COV and gradient of the ASL dataset were greater than the COV and gradient of perfusion (COV(ASL) = 1.64, COV(Q) = 1.01; gradient(ASL) = −2.56 × 10^−4^ mL/cm, gradient(Q) = −2.53 × 10^−4^ mL/cm). Filtering to 60% showed the best match between COV(ASL) and COV(Q) (0.99 and 1.01, respectively) and gradient(ASL) and gradient(Q) (−2.52 ×  10^−4^ mL/cm and −2.53 × 10^−4^ mL/ m, respectively) while filtering to 35% further decreased the COV and gradient of the ASL representation below that of perfusion (COV(ASL) = 0.86; COV(Q) = 1.01; gradient(ASL) = −2.30 × 10^−4^ mL/cm, gradient(Q) = −2.53 × 10^−4^ mL/cm) indicating removal of higher values of perfusion signal.

#### Prone versus supine – do we need to alter the filtering threshold?

In Figure 5A and B, when posture was changed from prone to supine, there was an increase in both the COV and gradient of blood (prone (COV = 1.09, gradient = −5.2 × 10^−03^ mL/cm); supine (COV = 1.18, gradient = −6.7 × 10^−03^ mL/cm). Figure 5C demonstrates the optimal threshold values for each posture. Although the optimal threshold value was generally higher for supine than prone posture, the difference in optimal threshold was observed to be within ~10%. Furthermore, both postures displayed lower per‐slice threshold values for the lateral slice (slice 1; (supine = 35%, prone = 30%)) than the medial slice (slice 5; (supine = 60%, prone = 65%).

### Impact of cardiac output

**Figure 6:**
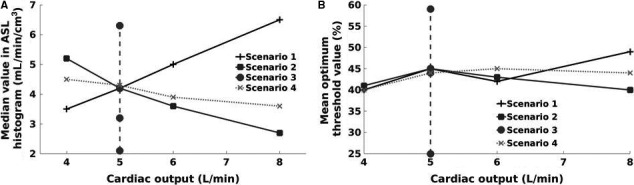
(A) Median ASL value from ASL frequency histogram; (B) Optimal threshold values for four different scenarios.

Figure 6 shows the effect of changes in heart rate (HR) and stroke volume (SV), where cardiac output (CO) = HR × SV, on the mean value from the ASL histogram (Fig. 6A) and the mean optimum threshold value (Fig. 6B). In Figure 6A, there is an interesting difference between varying SV and varying HR with respect to how increase in cardiac output affects per‐slice ASL signal. In varying SV, increase in cardiac output due to an increase in SV resulted in increased ASL (because more blood moved into the slice) while that resulting from an increase in HR resulted in decreased ASL due to shorter TI (and therefore more blood being in the “gap”). Furthermore, for varying HR and SV (which is normally observed), an increase in cardiac output resulted in a slight decrease in ASL since the increase in cardiac output was more from HR than SV. For constant CO, however, as HR increased ASL fell (as more blood originated from within the “gap”). In Figure 6B, when cardiac output increased from 4 to 8 L/min (varying SV, varying HR and varying HR and SV), the difference in the mean optimum threshold value remained within a 10% range.

## Discussion

### Relevance of study

ASL MRI is a quantitative imaging technique whereby blood is used as an endogenous tracer to measure the volume of blood flowing into a particular imaging plane. This method has been applied extensively for measurement of blood flow in the brain (Buxton 2005) and, more recently, has been applied in several physiological studies of the lung (Bolar et al. 2006; Henderson et al. 2009). From this extended analysis of the studies by Burrowes et al. 2012, the application of a single‐intensity threshold value of 35% was concluded to provide satisfactory removal of conduit vessel signal. Results from this study demonstrated that this value could be optimized for different conditions; however, the benefits of this fine tuning would be minimal.

### The intensity thresholding optimization cost function

The challenges with using the per‐slice total ASL signals in the estimation of per‐slice threshold value (Burrowes et al. 2012) is that it makes it difficult to independently assess changes to statistical metrics of the underlying perfusion signals. Therefore, in this study, an intensity thresholding optimization cost function was developed using statistical metrics of the individual components of the ASL signals (that is both perfusion and conduit vessel signals). The optimization cost function was made up of five parameters namely perfusion fraction, perfusion remaining after thresholding, conduit signals removed after thresholding, difference in gradient between the ASL signal and the underlying perfusion and difference in COV between the ASL signal and the underlying perfusion. These parameters were chosen because ASL ideally seeks to quantify perfusion signals, hence minimizing signal contribution from conduit vessels will improve on the fraction of perfusion signals within the voxels enabling a better match in gradient and COV to the underlying perfusion signals although this comes with some loss of perfusion signal. From Figure 2A and B, COV of the ASL signal best matched that of the underlying perfusion for both the lateral and medial slice at a threshold of 60% compared to the other threshold values. However, in Figure 2A, the gradient of the ASL signal matched that of the underlying perfusion signal at a lower threshold (30%) than that for the medial slice (80%). Furthermore, from Figure 3C and D as voxels with the highest signals (intensity = 100%) were removed from the slice there was a steeper increase in the amount of conduit vessel signals removed and perfusion fraction for the medial slice than the lateral. This is because of the higher number of large vessels in the medial region than the lateral region as seen by the hot spots in Figure 3C. Also, from Figure 2C and D, as high‐intensity voxels were eliminated from the slice there were losses in perfusion signals with these loses becoming greater at threshold values less than ~30%. Furthermore, below the intensity threshold value of ~30% per‐slice perfusion fraction did not change greatly (~2%) hence the intensity thresholding values above 30% were considered appropriate. The cost function predicted optimal threshold values were based on equation (3); however, depending on which parameters are included in this cost function and their respective weights, this would be predicted to result in different outcomes. Furthermore, predicting an exact value is difficult to do in the in‐vivo imaging reality, therefore what this study shows is that while there is a degree of variation in the optimal value, the differences are relatively small and likely inconsequential.

### Do we need to change threshold as a function of slice?

When slice location is changed from the peripheral to the medial regions of the lungs, the signal within a slice increases due to the predominance of large vessels in the medial regions (Fig. 3B) of the lungs (Burrowes et al. 2012) with a corresponding lower proportion of perfusion signal. The optimum threshold values proposed for the lateral slices (~30%, Fig. 3D) were similar to that proposed in previous study (Burrowes et al. 2012) for a cardiac output of 5000 mL/min. The model however proposed higher threshold values for minimizing non‐capillary blood signals within the medial slices (~60%). This was expected since from Figure 2C and D, after removing 5% of the highest intensity voxels, ∆COV was observed to influence the cost function the most with a minimum value (suggesting a better match in COV between the remaining ASL signal and the underlying perfusion) at an intensity threshold value of 60%. Furthermore, a comparison of the cost function values for both slices 4 and 5 (shown in Fig. 1) showed that in the range of about 35% to 75%, the cost function is flat and near its minimum, so anywhere in that range is a reasonable choice. This study then examined and compared the effects of using both the standard thresholding value of 35% and the 60% predicted by the model in minimizing non‐capillary blood in the medial slice as shown in Figure 4. Although filtering by an intensity threshold value of 60% provided a marginally better match in gradient and COV (of the remaining ASL signal and underlying perfusion) than 35%, the percentage change in the underlying perfusion was only ~5%. Also, there was an improved fraction of perfusion signals within the remaining ASL signals (~78%) when the 35% thresholding value is used. Interestingly, from the two curves, we observed a steeper change in the cost function value below an intensity threshold value of ~35% (corresponding to what is normally used in ASL MRI) which also corresponded to the value below which there was a steeper loss in the underlying perfusion signals and concluded that using an intensity threshold value of 35% will provide an adequate estimate of the amount of perfusion signals to be quantified within the ASL signals, and brings with it the advantage of having a uniform threshold value regardless of slice position.

### Prone versus supine – do we need to alter the filtering threshold?

From Figure 5A, when posture was changed from supine to prone the mean ASL signals within the slice increased slightly due to the slight increase in cardiac output that occurs as seen in a previous study (Rohdin et al. 2003). That is, if the left atrial pressure is kept constant, then an increase in cardiac output (assuming HR is constant) will correspond to an increase in the flow rate of blood; hence the increase in the mean of the ASL signals observed for that slice. Furthermore, in Figure 5B, a statistically significant reduction in COV (*P* = 0.03) was observed when posture was changed from supine to prone (1.17–1.08). This observation (decrease in COV) is consistent with studies by (Glenny et al. 1991)(COV = 0.44 supine, COV = 0.39 prone), (Chon et al. 2006)(COV=0.32 supine, COV=0.28 prone), and (Prisk et al. 2008)(COV = 0.71 supine, COV = 0.70 prone) although their studies involved only blood flow in the capillaries and their decrease in COV of capillary blood did not achieve statistical significance. The high COV values predicted by the model used in this study are expected as flow in large vessels was considered as well. Also, from Figure 5B, when posture was changed from supine to prone, a decrease in the gradient of blood (supine: gradient = −6.7 × 10^−03^ mL/cm; prone: gradient = −5.2 × 10^−03^ mL/cm) was recorded although not statistically significant (*P* = 0.44). Similar observation (decrease in gradient when posture change from supine to prone) was recorded by Prisk et al. 2008 although the decrease in gradient in their analysis achieved statistical significance. Furthermore, in Figure 5C, although the optimum threshold value for supine was generally higher than that for prone the difference was observed to be not significant (*P* = 0.55; *α* = 5%) Therefore, we concluded that the optimum intensity threshold value for a slice can be considered to be independent of posture.

### Impact of cardiac output

In assessing the effects of cardiac output on per‐slice ASL signals and the proposed thresholding methodology, four scenarios were used as shown in Figure 6. For constant cardiac output (5 L/min), HR was varied while cardiac output ranging from 4 to 8 L/min was obtained by varying SV or varying HR or varying SV and HR. When cardiac output was kept constant, increasing HR (decreasing TI) resulted in a decrease in per‐slice ASL signals as the probability of blood originating from the “gap” increases. The optimum thresholding value also decreased with increase in HR because of the reduced signals within the slice. Simultaneous increase in HR and SV best represents cases involving increase in cardiac output. Normally increase in cardiac output results from both increase in HR and SV although the rate of increase in HR is higher than that of SV. In this approach, a slight decrease in ASL signals from 4.6 to 3.6 mL/min/cm^3^ was observed because of the higher impact of HR than SV. The median ASL signals were however observed to be within 5% when cardiac output increased from 4 mL/min to 8 L/min. For varying SV or varying HR, two extreme cases for increase in cardiac output were tested. Interestingly, while for varying SV, an increase in cardiac output (resulting from increase in SV) caused an increase in both the ASL signals and the optimum thresholding value, while varying HR increase in cardiac output (resulting from increase in HR) caused a decrease in both ASL signals and the optimum thresholding value. This is because for varying SV (and for a fixed HR), an increase in SV means more blood entering the slice and hence an increase in both the ASL signals and optimum thresholding value. However, for varying HR (and for a fixed SV), an increase in HR increases the probability of blood originating from the “gap,” and hence a decrease in ASL and optimum thresholding value. If the very high‐cardiac output (8 L/min) is neglected, the range of mean optimum threshold value was between 40 and 45%; hence using the standard thresholding value of 35% was considered appropriate.

## Conclusions

This study aimed to characterize the behavior of an intensity thresholding methodology for varying conditions of slice location, posture, and cardiac output using the individual components of the ASL signal (capillary, arterial, and venous). Previously developed models of the pulmonary circulation and ASL were used to predict blood flow in the pulmonary circulation and the corresponding ASL MRI quantification of perfusion. The results of this study showed an increase in both the total ASL signal and heterogeneity (COV = 0.90 to COV = 1.65) of the ASL signal as slice location changed from lateral to medial due to the dominance of large vessels in the medial regions of the lung. Also changing the lung posture from supine to prone was found to result in a decrease in both the gradient and COV of blood (supine: COV = 1.18, gradient = −6.7 × 10^−03^ mL/cm; prone: COV = 1.09, gradient = −5.2 × 10^−03^ mL/cm); while the decrease in COV was statistically significant (*P* = 0.03, *α* = 5%), the decrease in gradient was not statistically significant (*P* = 0.44, *α* = 5%). Furthermore, an increase in cardiac output resulting mainly from SV was observed to increase the median of the ASL signals (due to the increase in blood volume within each voxel) while that resulting mainly from HR decreased the median of the ASL signals (due to an increase in the probability of blood originating from the gap). Although the cost function analysis predicted a 60% threshold value for reducing large vessel signals in the medial regions of the lung, application of a threshold value of 35% also provided a good result with regard to gradient, COV, and perfusion fraction. Finally, this study determined that the intensity threshold value for a slice is independent of slice location, posture, and cardiac output.

### Limitation

This study is modeling‐based and did not involve the use of real imaging data. Furthermore, only a healthy subject is considered hence TI corresponding to 80% of RR was adequate. The blood flow solutions, T1 and T2 used were representatives of a healthy human lung. It is also possible to develop the human lung model to mimic abnormal conditions and subsequently simulate the flow of blood through it within our modeling; however, this is outside of the scope of the current study. In addition, the intensity cost function has not been tested for unhealthy subjects. In developing the ASL model, the ASL MRI protocol was simplified by neglecting all tissues and using just the blood flow within the pulmonary vessels; hence correction for receiver coil sensitivity associated with depth within tissues was not considered.
